# Supplementary material for: Presenilin-mediated cleavage of APP regulates synaptotagmin-7 and presynaptic plasticity
Source: Nat Commun. 2018 Nov 14;9:4780. doi: 10.1038/s41467-018-06813-x (PMC6235831; doi:10.1038/s41467-018-06813-x)
Supplement: Supplementary file 2 — Supplementary Information [file 41467_2018_6813_MOESM2_ESM.pdf]

Reviewers' comments:

Reviewer #1 (Remarks to the Author):

This is an interesting and potentially important paper, but it is not convincing in its present form. I have a couple of major concerns.
1.Could differences in excitability for the different conditions contribute to an apparent alteration in facilitation? The light activation experiments of supplementary Figure 1 seem too good to be true and represent an incomplete characterization of light activation for the study of synaptic transmission. It would be useful to see what the reliability of firing was like as a function of light intensity. What does it look like for a range of intensities? Are the thresholds for action potential initiation different for different cells in the same preparation are for cells in different slices from different animals. Light intensity data is only provided for synaptic transmission for 0.1 Hz stimulation. It should also be provided for granule cell action potential generation. Also, are there failures to excite, are there 2 spikes evoked per stimulus and do cells ever go into depolarization block? These experiments have only been performed for control conditions and the reliability of stimulation has not been examined under conditions where facilitation is altered. These are important experiments.
2. I am concerned that expression of Syt7 in wildtype animals would also increase frequency dependent facilitation. If this would true it would make the apparent rescue of facilitation more difficult to interpret. The expression levels of Syt7 in rescue studies is also important. Do they express at comparable levels to control conditions or at much higher levels.
3. They need to provide a measure of the initial probability of release for their experimental conditions to establish that it is a direct alteration in synaptic plasticity rather than a secondary effect on facilitation as a result of an increase in the probability of release.
4. Are these results restricted to mossy fibers. What happens if similar experiments are performed at CA3 to CA1 synapses?

Minor points:
Line 192 It seems premature to say Syt7 deficit is specific with so few proteins examined. The statement should be softened.

Reviewer #2 (Remarks to the Author):

In this paper the authors show that genetic deletion of presynaptic Presenilin 1 and 2 reduces axonal levels of synaptotagmin-7 and impairs synaptic facilitation and replenishment of release-competent synaptic vesicles. In addition, they show that the Synaptotagmin-7 levels are post-transcriptionally regulated and dependent on γ-secretase activity. Further, the absence of both APP and Presenilin reverses the loss of axonal Synaptotagmin-7. The work suggests that synaptic vesicle processing may affected in Alzheimer’s disease.
This paper is impressive in the methods and techniques that are used to analyze the problem (eg. Laser assisted microdissection, combination of cell-specific optogenetic

activation with gene deletion or rescue) as well as in the overall message, that synaptic vesicle mechanisms are involved in AD. The downside is that the molecular mechanism involving the substrates of PS are less strongly supported by data as pointed out below. However, I believe that these issues can be addressed in a revised manuscript.

Major comments

1- The authors propose a mechanism where blocking Presinilin-mediated cleavage of APP results in the accumulation of APP-CTF, leading to the sequestration and degradation of Synaptotagmin-7. Although they show that subsequent to depletion of PS the levels of APP-CTF increase and levels of Synaptotagmin-7 decrease in the presynaptic compartment, the evidence supporting a functional connection between these two processes is lacking. An interaction between APP-CTF and Syt7 is solely shown by a co-immunoprecipitation experiment on neuronal lysates where the appropriate controls are lacking (showing that APP and APP-CTF do not simply precipitate together with the beads or the antibody used for the IP of Syt7). The observation that APP-CTF and Syt7 can interact does not mean they do so inside the neuron and specifically in the presynaptic compartment. The paper would benefit from further strengthening this central claim of the paper, eg by testing if the proteins colocalize in their neuronal cultures, as well as using other experiments.

2- the authors suggest that PS knock out-induced decrease of Syt7 causes an impairment in synaptic plasticity, shown by impaired facilitation and SV replenishment in PSKO mice. The connection between the loss of PS and the impairment in synaptic plasticity is not fully shown: (a) the authors claim that PS knock out mice show 'unaltered synaptic terminals' (page 6, line 143), a result supported by the quantification of the number and size of the boutons from mossy fibers terminals. However, this quantification is based on 3D reconstructions of confocal images where it is difficult to define Bassoon puncta (Supplementary Fig 2g). To more convincingly show that the loss of PS does not alter the morphology of the synaptic terminals, it would be better to include electron microscopic analyses.

(b) A related concern is that the authors do not find a complete rescue of SV replenishment by re-expressing Syt7 in PSKO mice; although the rate of SV replenishment is not significantly different from that of wild type mice. How about the comparison to PSKO mice? (Figure 4f).

3- It is not clear what the roles of Presinilin 1 and Presinilin 2 are in relation to the reported presynaptic effects. According to the data presented, the deletion of presynaptic PS1 alone seems to be sufficient to cause a deficit in synaptic plasticity (Supplementary Fig. 2a) but is not sufficient to downregulate the protein levels of Syt7 to the wild type level (Fig. 3c and Fig 7c). This divergence suggests that the deficits in synaptic plasticity and the reduction of Syt7 are not similarly regulated by PS1 and PS2, although there might be a functional connection. The authors could address this using the independent PS 1 and PS2 deficient mice they have access to, allowing them to elucidate the specific functions of PS and PS2. Without such analyses, speculation about possible compensatory mechanisms between the two paralogs (page 10 line 279) may not be fully appropriate.

Minor comments

-"We discovered that Ps controls the expression of the Ca^{2+} sensor Syt7 presynaptically." I think the better word choice here is "presence" instead of "expression".

-In figure 4f WT and Syt7 rescue are statistically compared. What is missing here is the comparison between PSKO and Syt7 rescue.
-To test the involvement of AICD in presynaptic mechanisms the authors express AICD in PSKO GCs and show no effect on facilitation and basal transmission. However, the authors did not show the expression of AICD in the GCs. The absence of an effect could be a result of a lack of AICD expression in the GCs.
- It would be useful to assess the effect of a single APPKO on Syt7 levels.

Reviewer #3 (Remarks to the Author):

In this submission, Barthet and colleagues investigate the function of presenilins in presynaptic plasticity. The authors utilized lentiviral gene expression to selectively ablate PS1 expression in PS2 KO mice and take advantage of an optogenetic strategy to selectively activate dentate gyrus granule cells. This allowed them to ascertain how the loss of presenilin expression in presynaptic granule cells affect the functional properties of mossy fiber-CA3 synapses. By extensive characterization of mossy fiber-EPSCs evoked in CA3 pyramidal cells, the authors found that presenilin expression is essential for presynaptic facilitation. The defect was traced to the synaptic vesicle replenishment rate at the mossy fiber-CA3 synapses in the absence of morphological alterations of the terminals. The authors next addressed the underlying molecular mechanism and found a decrease in the levels of the Ca2+ sensor synaptotagmin 7 in the stratum lucidum. Lentiviral overexpression of synaptotagmin 7 rescued the functional phenotype in PSKO mice. The authors next analyzed synaptotagmin 7 levels in PSKO mice and cultured neurons treated with gamma-secretase inhibitors to reveal a causal relationship between the accumulation of APP C-terminal fragments and a reduction in synaptotagmin 7 abundance.

This study details compelling evidence that gamma-secretase processing of APP in dentate gyrate granule cells regulates synaptotagmin 7 levels and presynaptic plasticity. There is an excellent implementation of the optogenetic strategy to combine selective gene deletion in dentate gyrus granule cells with cell-specific activation to characterize the properties of mossy fiber-CA3 synapses. Overall, the experiments are performed with proper controls and the results are interpreted appropriately. The findings are novel and will be interesting for the readership.

Specific points are listed below.

1) The authors have not attempted to quantify the extent of reduction in presenilin 1 expression.
2) The evidence for successful AICD expression should be included as part of Fig. 5i in order to accurately interpret the negative findings on AICD.

Reviewer Comments:

Reviewer #1:

This is an interesting and potentially important paper, but it is not convincing in its present form. I have a couple of major concerns.

***1.** Could differences in excitability for the different conditions contribute to an apparent alteration in facilitation? The light activation experiments of supplementary Figure 1 seem too good to be true and represent an incomplete characterization of light activation for the study of synaptic transmission. It would be useful to see what the reliability of firing was like as a function of light intensity. What does it look like for a range of intensities? Are the thresholds for action potential initiation different for different cells in the same preparation are for cells in different slices from different animals. Light intensity data is only provided for synaptic transmission for 0.1 Hz stimulation. It should also be provided for granule cell action potential generation. Also, are there failures to excite, are there 2 spikes evoked per stimulus and do cells ever go into depolarization block? These experiments have only been performed for control conditions and the reliability of stimulation has not been examined under conditions where facilitation is altered. These are important experiments.*

We agree with the reviewer that differences in GC excitability between WT and PSKO genotype could, at least in part, account for the differences in synaptic transmission observed between the two conditions. As recommended by the reviewer, we examined the reliability of firing as a function of light intensity and we measured the threshold for action potential firing of GCs. We also measured the amplitude and the half-width of the action potentials, two parameters that could impact synaptic transmission. As requested, we performed these analyses in the two genotypes (23 cells each) and at two frequencies of stimulation.

1) The reliability of firing as a function of light intensity was similar between WT and PSKO. At 20% of light intensity, 78% of the ChIEFtom-positive GCs were firing. At 100% light intensity, 100% of the ChIEFtom positive GCs were firing. Each individual GC respond in an all-or-none manner in relation to light intensity: below their individual threshold to light responsiveness, they do not fire; above they fire without failure. All the experiments of synaptic transmission in the present study have been performed at 100% light intensity.

2) For single action potentials, we observed no difference in the threshold (-40 mV), amplitude (118 mV) and half-width (1.8 ms) of APs between the two genotypes.

3) During trains of 10 stimuli at 20 Hz, the amplitude of the APs decreased whereas their threshold and their half-width increased as a function of stimulus number. Yet, no difference between genotypes was observed.

4) We paid attention to the presence of double spikes. We only observed these in one WT- and one PSKO-GC. For these two cells, the second spikes disappeared during trains after the second stimulus.

5) Using 1ms-light pulse to activate ChIEF, we did not observe any depolarization block even during trains. This contrasts with the observation of depolarization block when long and square current is injected in the GC via the patch pipette.

Seven new sub-figures have been added in supplementary Figure S1 (h,i) and S2 (b). They bring valuable information about GC firing and about the reliability of this optogenetic tool to study the synaptic role of PS as described in this study. We would like to thank the reviewer for his recommendations.

***2.** I am concerned that **a)** expression of Syt7 in wildtype animals would also increase frequency dependent facilitation. If this would true it would make the apparent rescue of facilitation more difficult to interpret. **b)** The expression levels of Syt7 in rescue studies is also important. Do they express at comparable levels to control conditions or at much higher levels.*

a) We performed a new set of synaptic transmission experiments with a new condition (WT + Syt7) to address the point raised by the reviewer. We found that over-expression of Syt7 in WT does not enhance presynaptic facilitation (new result). Together with restored presynaptic facilitation in PSKO synapses

by re-expression of Syt7, this supports our conclusion that Syt7 is a molecular link between PS and presynaptic facilitation.

b) The rescue experiment of this paper has involved a virus with a synapsin promoter, a strategy commonly used in recent works. The expression level of Syt7 does not perfectly match WT levels, as the promoter is not the full length promoter of Syt7. However the experiment in a) proposed by the reviewer shows that this expression level does not promote artificial facilitation in WT.

3. *They need to provide a measure of the initial probability of release for their experimental conditions to establish that it is a direct alteration in synaptic plasticity rather than a secondary effect on facilitation as a result of an increase in the probability of release.*

We agree with the referee that the relationship between presynaptic facilitation and initial probability of release is important. Providing a direct measure of initial release probability at Mf synapses is very challenging (e.g. multiple release sites).

We feel however that the original article already contained multiple piece of information indicating that the initial probability of release is comparable between the two genotypes. Most importantly, the amplitude and the failure rate at 0.1 Hz are not statistically different between WT and PSKO, and this is paralleled by a lack of difference in the quantal size (Cd^{2+} experiments). In addition we found no difference in paired pulse facilitation. Importantly, the volume of individual MfBs and the overall levels of major presynaptic proteins do not support a marked change in the number of release sites, a parameter which could have confounded the comparison of basal synaptic properties. We have added new analysis further supporting that the initial probability of release is comparable between genotypes. Indeed, we observed that the variability of the amplitude of the EPSC, assessed by the coefficient of variability (C.V.) is similar between WT and PSKO (Fig. S1l). Altogether our results do not provide any evidence for an increase in the initial probability of release (at 0.1 Hz).

Nonetheless, we attempted to perform multiple-probability fluctuation analysis (MPFA; Silver 2003) to obtain an estimation of initial Pr. This required to increase Pr (by increasing extracellular Ca^{2+}) in order to acquire a variance-mean plot that can be fitted with a parabola. Under our experimental conditions (optogenetic stimulation), we were not able to draw a full parabola.

4. *Are these results restricted to mossy fibers. What happens if similar experiments are performed at CA3 to CA1 synapses?*

We agree with the referee that the comparison with different synapses is interesting. The comparison between facilitating and depressing synapses, between glutamatergic and inhibitory synapses would indeed provide valuable information on the synaptic roles of PS and Syt7. The amount of work is however considerable and we consider that it will constitute a follow-up study on its own.

Minor points:

Line 192 It seems premature to say Syt7 deficit is specific with so few proteins examined. The statement should be softened.

We softened this statement.

Reviewer #2 :

In this paper the authors show that genetic deletion of presynaptic Presenilin 1 and 2 reduces axonal levels of synaptotagmin-7 and impairs synaptic facilitation and replenishment of release-competent synaptic vesicles. In addition, they show that the Synaptotagmin-7 levels are post-transcriptionally regulated and dependent on γ-secretase activity. Further, the absence of both APP and Presenilin reverses the loss of axonal Synaptotagmin-7. The work suggests that synaptic vesicle processing may affected in Alzheimer's disease.

This paper is impressive in the methods and techniques that are used to analyze the problem (eg. Laser assisted microdissection, combination of cell-specific optogenetic activation with gene deletion or rescue) as well as in the overall message, that synaptic vesicle mechanisms are involved in AD. The downside is that the molecular mechanism involving the substrates of PS are less strongly supported by data as pointed out below. However, I believe that these issues can be addressed in a revised manuscript.

First, we would like to thank the referee for his positive evaluation of our study.

Major comments

1- *The authors propose a mechanism where blocking presenilin-mediated cleavage of APP results in the accumulation of APP-CTF, leading to the sequestration and degradation of Synaptotagmin-7. Although they show that subsequent to depletion of PS the levels of APP-CTF increase and levels of Synaptotagmin-7 decrease in the presynaptic compartment, the evidence supporting a functional connection between these two processes is lacking. An interaction between APP-CTF and Syt7 is solely shown by a co-immunoprecipitation experiment on neuronal lysates where the appropriate controls are lacking (showing that APP and APP-CTF do not simply precipitate together with the beads or the antibody used for the IP of Syt7). The observation that APP-CTF and Syt7 can interact does not mean they do so inside the neuron and specifically in the presynaptic compartment. The paper would benefit from further strengthening this central claim of the paper, eg by testing if the proteins colocalize in their neuronal cultures, as well as using other experiments.*

We agree with the reviewer about the importance of studying whether the interaction between APP and Syt7 occurs in presynaptic compartments. To address the reviewer's points, we performed two new sets of experiments, one based on cell fractionation, the second based on immunostaining.

1) We tested the interaction between Syt7 and APP in synaptosomal lysates prepared from adult mouse brains. First, the characterization of the synaptosomal preparation informed us about the relative expression level of the different proteins studied here. Our synaptosomal fraction was characterized by a robust enrichment in the active zone proteins SNAP-25 and syntaxin-1 whereas the synaptic vesicle protein Vamp2 was only moderately enriched. This suggested that our synaptosomal preparation was more enriched in presynaptic terminals membranes than in synaptic vesicles. In such a synaptosomal fraction, Syt7 was enriched in a manner similar to Vamp2, in agreement with previous Fig7d now 7e (cell fractionation on iodixanol gradient) and with an expression in synaptic vesicles. APP, both full-length and CTFs, were found in synaptosomes in agreement with the literature (Laßek, M. et al. 2013; Wilhelm, B. G. et al. 2014). PS1 was also present in synaptosomes but not enriched in comparison with the homogenate. These new data have been added as supplementary FigS7a.

Using synaptosomes as a starting material, we succeeded to co-IP APP when pulling down Syt7 (Synaptic Systems rabbit antibody #105173). Importantly, we also succeeded with the reverse co-IP, i.e. to co-IP Syt7 when pulling down APP with a C-terminal antibody (Y188 rabbit antibody Abcam #32136). This clearly indicates that the two proteins are part of presynaptic protein complexes. As recommended by the referee, we added the proper controls to this new set of experiments. These new results have been added as main Fig7d.

For this reverse co-IP, we used a new antibody against Syt7 to clarify the WB detection: the mouse antibody, clone N275/14 from Millipore which recognizes an epitope (within C2A domain) different than the antibody against Syt7 from Sysy. We characterized this antibody by staining hippocampal sections from WT and Mf/PSKO condition. Notably, we could reproduce the main results of this study, i.e. the dramatic decreased in the expression of Syt7 in PSKO Mf (as in previous Fig3a). We added this new data in supplementary FigS3.

2) We investigated the possible co-localization between APP and Syt7 in neuronal cultures. By labelling APP in green and Syt7 in red and with a pixel size of 120 nm, we measured a Manders coefficient of 0.77 in control conditions. Interestingly, the Manders coefficient increased to 0.94 when the neurons were treated with GSI suggesting than the accumulated APP-CTFs colocalize with Syt7. Notably the

reverse staining with APP in red and Syt7 in green gave similar results. This experiment is illustrated in FigS7b.

In total, four new sub-figures have been added to further support the interaction between APP and Syt7. They bring valuable information about the localization and the extent of this interaction. They consolidate the mechanistic part of this study. We would like to thank the reviewer for his recommendation.

***2-** the authors suggest that PS knock out-induced decrease of Syt7 causes an impairment in synaptic plasticity, shown by impaired facilitation and SV replenishment in PSKO mice. The connection between the loss of PS and the impairment in synaptic plasticity is not fully shown:*

(a) the authors claim that PS knock out mice show 'unaltered synaptic terminals' (page 6, line 143), a result supported by the quantification of the number and size of the boutons from mossy fibers terminals. However, this quantification is based on 3D reconstructions of confocal images where it is difficult to define Bassoon puncta (Supplementary Fig 2g). To more convincingly show that the loss of PS does not alter the morphology of the synaptic terminals, it would be better to include electron microscopic analyses.

We agree with the reviewer that 3D-reconstruction with serial EM is a superior technique to study the morphology of complex object like Mf terminals. Unfortunately, this is a technical expertise not available in the lab or in the institute. We contacted Cordelia Imig (Brose lab) to collaborate and study Mf morphology and content (number of docked vesicles…) in WT and PSKO. However, the amount of work appeared considerable and not realizable within a reasonable time-frame.

The original article already contained multiple piece of information indicating that the morphology/protein content of MfBs is comparable between the two genotypes. Indeed, four approaches were employed: 1) 3D reconstruction of MfBs using confocal microscopy (FigS2f), 2) STED microscopy of Bassoon stainings (FigS2g), 3) confocal acquisition of three presynaptic markers (Fig3f,g), 4) Western-blotting of three presynaptic markers (Fig5b,d).

(b) A related concern is that the authors do not find a complete rescue of SV replenishment by re-expressing Syt7 in PSKO mice; although the rate of SV replenishment is not significantly different from that of wild type mice. How about the comparison to PSKO mice? (Figure 4f).

The original Fig4f involved a lower number of cells compared to other groups possibly impacting the statistical power. We performed a new set of experiment with n≥18 per group. The "PSKO + Syt7" group is statistically different compared to the PSKO group.

***3-** It is not clear what the roles of Presenilin 1 and Presenilin 2 are in relation to the reported presynaptic effects. According to the data presented, the deletion of presynaptic PS1 alone seems to be sufficient to cause a deficit in synaptic plasticity (Supplementary Fig. 2a) but is not sufficient to downregulate the protein levels of Syt7 to the wild type level (Fig. 3c and Fig 7c). This divergence suggests that the deficits in synaptic plasticity and the reduction of Syt7 are not similarly regulated by PS1 and PS2, although there might be a functional connection. The authors could address this using the independent PS 1 and PS2 deficient mice they have access to, allowing them to elucidate the specific functions of PS and PS2. Without such analyses, speculation about possible compensatory mechanisms between the two paralogs (page 10 line 279) may not be fully appropriate.*

We agree that the compensatory mechanisms we proposed in the original version was speculative. We truncated the part of sentence mentioning it.

Minor comments

-"We discovered that Ps controls the expression of the Ca2+ sensor Syt7 presynaptically." I think the better word choice here is "presence" instead of "expression".

We have replaced "expression" by "levels".

-In figure 4f WT and Syt7 rescue are statistically compared. What is missing here is the comparison between PSKO and Syt7 rescue.

Done. See point 2b.

-To test the involvement of AICD in presynaptic mechanisms the authors express AICD in PSKO GCs and show no effect on facilitation and basal transmission. However, the authors did not show the expression of AICD in the GCs. The absence of an effect could be a result of a lack of AICD expression in the GCs.

We have added WB experiments showing the expression of AICD using this construct in Fig.S5.

- It would be useful to assess the effect of a single APPKO on Syt7 levels.

We are currently studying the physiological role of APP in presynaptic mechanisms using APPfloxed mouse line and following an approach very similar to the one presented here. The results will be presented in an independent article.

Reviewer #3:

In this submission, Barthet and colleagues investigate the function of presenilins in presynaptic plasticity. The authors utilized lentiviral gene expression to selectively ablate PS1 expression in PS2 KO mice and take advantage of an optogenetic strategy to selectively activate dentate gyrus granule cells. This allowed them to ascertain how the loss of presenilin expression in presynaptic granule cells affect the functional properties of mossy fiber-CA3 synapses. By extensive characterization of mossy fiber-EPSCs evoked in CA3 pyramidal cells, the authors found that presenilin expression is essential for presynaptic facilitation. The defect was traced to the synaptic vesicle replenishment rate at the mossy fiber-CA3 synapses in the absence of morphological alterations of the terminals. The authors next addressed the underlying molecular mechanism and found a decrease in the levels of the Ca2+ sensor synaptotagmin 7 in the stratum lucidum. Lentiviral overexpression of synaptotagmin 7 rescued the functional phenotype in PSKO mice. The authors next analyzed synaptotagmin 7 levels in PSKO mice and cultured neurons treated with gamma-secretase inhibitors to reveal a causal relationship between the accumulation of APP C-terminal fragments and a reduction in synaptotagmin 7 abundance.

This study details compelling evidence that gamma-secretase processing of APP in dentate gyrate granule cells regulates synaptotagmin 7 levels and presynaptic plasticity. There is an excellent implementation of the optogenetic strategy to combine selective gene deletion in dentate gyrus granule cells with cell-specific activation to characterize the properties of mossy fiber-CA3 synapses. Overall, the experiments are performed with proper controls and the results are interpreted appropriately. The findings are novel and will be interesting for the readership.

First, we would like to thank the referee for his very positive and supportive evaluation of our study, from the optogenetic strategy to the evidence that gamma-secretase processing of APP regulates synaptotagmin 7 levels and presynaptic plasticity.

Specific points are listed below.

1) The authors have not attempted to quantify the extent of reduction in presenilin 1 expression.

Unfortunately, the antibodies against PS1 do not work for immunolabelling in brain sections. To demonstrate the reduction of PS1 protein levels upon genetic invalidation of PS1 gene, we used primary neuronal cultures from PS1floxed embryos. We infected them with viruses expressing either GFP or Cre-GFP under the synapsin promoter. We compared by WB the content in PS1 of these two conditions to a naïve neuronal culture. While the infection with the GFP expressing virus does not alter PS1 expression, the expression of Cre dramatically decreases the expression of PS1 protein.

2) The evidence for successful AICD expression should be included as part of Fig. 5i in order to accurately interpret the negative findings on AICD.

We added WB showing the expression of AICD using this construct.

REVIEWERS' COMMENTS:

Reviewer #1 (Remarks to the Author):

The authors have performed additional experiments and have addressed my primary concerns.

Reviewer #2 (Remarks to the Author):

I thank the authors for addressing my questions. I am largely convinced by the answers but in response to question 3 I would have liked to see the data obtained with PS1 and PS2 single KO animals.

"3- It is not clear what the roles of Presenilin 1 and Presenilin 2 are in relation to the reported presynaptic effects. According to the data presented, the deletion of presynaptic PS1 alone seems to
be sufficient to cause a deficit in synaptic plasticity (Supplementary Fig. 2a) but is not sufficient to downregulate the protein levels of Syt7 to the wild type level (Fig. 3c and Fig 7c). This divergence
suggests that the deficits in synaptic plasticity and the reduction of Syt7 are not similarly regulated by PS1 and PS2, although there might be a functional connection. The authors could address this using the independent PS 1 and PS2 deficient mice they have access to, allowing them to elucidate the specific functions of PS and PS2."

Reviewer #1 (Remarks to the Author):

The authors have performed additional experiments and have addressed my primary concerns.

We thank the reviewer for his suggestions and comments that helped improve the impact of the paper.

Reviewer #2 (Remarks to the Author):

I thank the authors for addressing my questions. I am largely convinced by the answers but in response to question 3 I would have liked to see the data obtained with PS1 and PS2 single KO animals.

"3- It is not clear what the roles of Presenilin 1 and Presenilin 2 are in relation to the reported presynaptic effects. According to the data presented, the deletion of presynaptic PS1 alone seems to be sufficient to cause a deficit in synaptic plasticity (Supplementary Fig. 2a) but is not sufficient to downregulate the protein levels of Syt7 to the wild type level (Fig. 3c and Fig 7c). This divergence suggests that the deficits in synaptic plasticity and the reduction of Syt7 are not similarly regulated by PS1 and PS2, although there might be a functional connection. The authors could address this using the independent PS 1 and PS2 deficient mice they have access to, allowing them to elucidate the specific functions of PS and PS2."

We thank the reviewer for his suggestions and comments that helped improve the impact of the paper. Unfortunately, the PS2 single KO mouse line is currently not available in our animal facility due to space restriction (we do have the frozen sperm though). While we agree with the referee that the suggested experiments is interesting, we cannot perform it within a reasonable amount of time.
