# Supplementary material for: Presenilin-mediated cleavage of APP regulates synaptotagmin-7 and presynaptic plasticity
Source: Nat Commun. 2018 Nov 14;9:4780. doi: 10.1038/s41467-018-06813-x (PMC6235831; doi:10.1038/s41467-018-06813-x)

Supplementary information

Presenilin-mediated cleavage of APP regulates synaptotagmin-7 and presynaptic plasticity

Barthet *et al.*

Supplementary table 1

Gene	GenBank ID	Forward Sequence (5’-3’)	Reverse Sequence (5’-3’)
Nono	NM_02314	CTGTCTGGTGCATTCCTGAACTAT	AGCTCTGAGTTCATTTTCCCATG
Ppia	NM_008907	CAAATGCTGGACCAAACACAA	GCCATCCAGCCATTCAGTCT
Syt7	NM_018801	GTGAGAAGAAAGCCATCAAGTT	CCGGGCACTGGGGCTGTA

Supplementary table 1 Mouse qPCR primer sequences

Supplementary figure 1

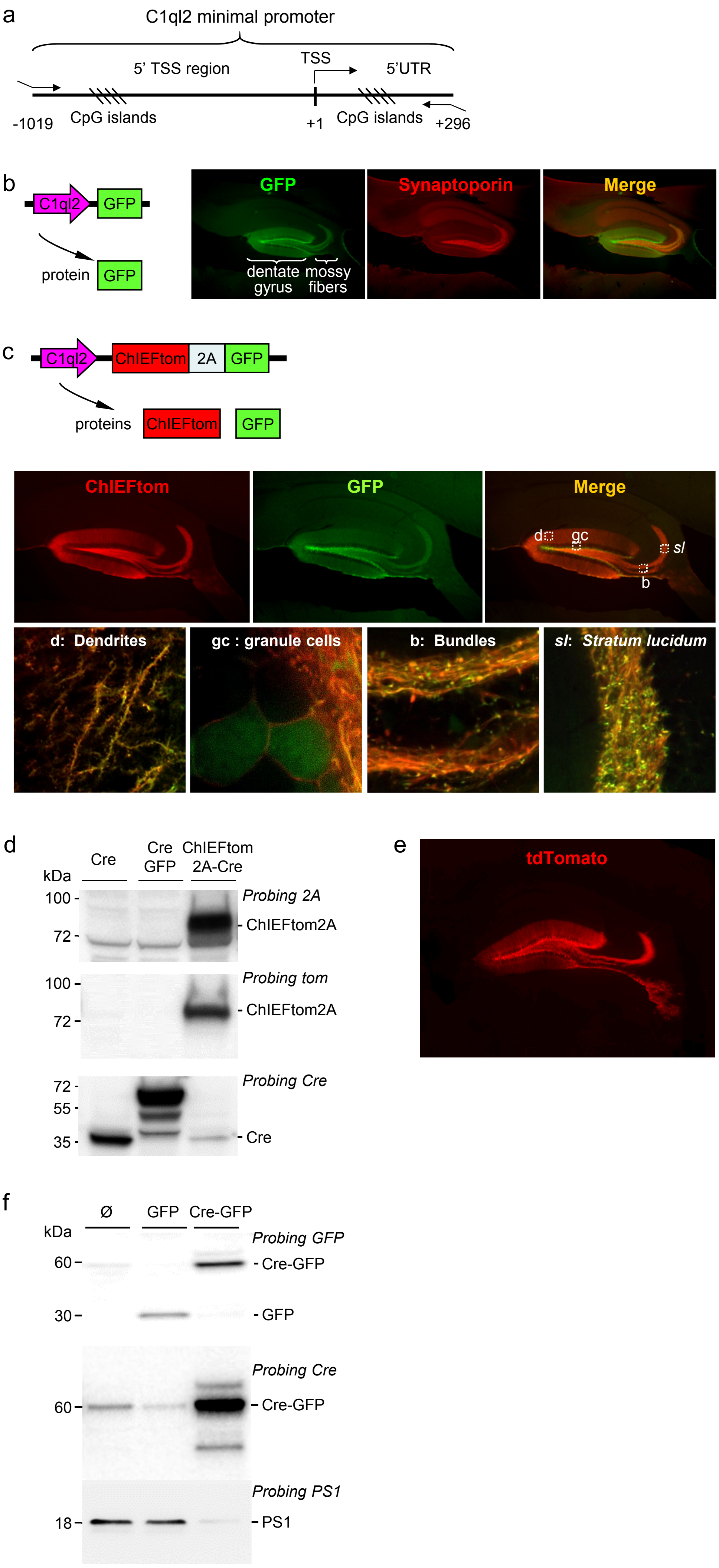

Supplementary figure 1

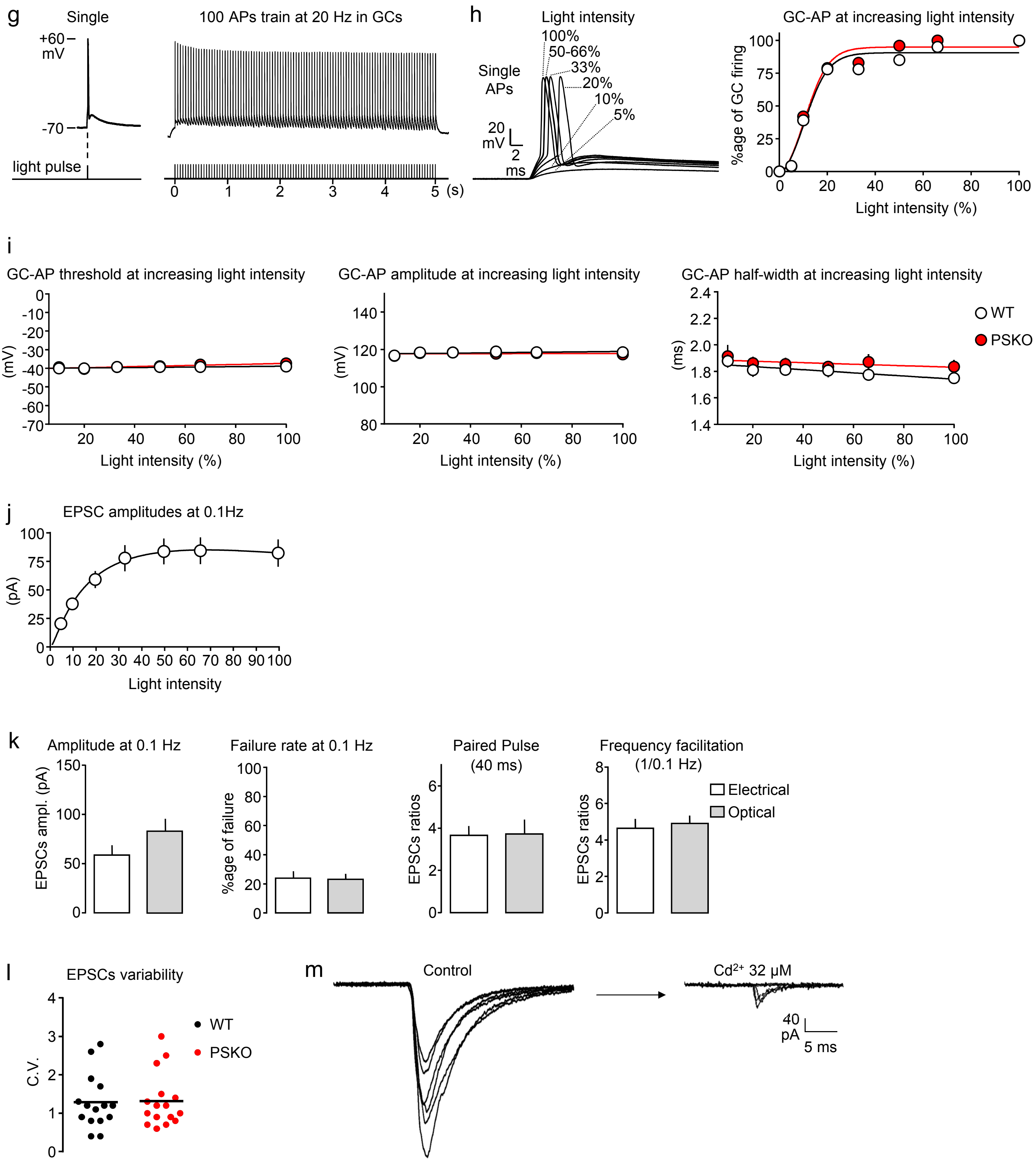

Supplementary figure 1 Combined DG-specific gene deletion with light activation. **a** Schematic representation of the mouse C1ql2 minimal promoter cloned from genomic DNA. The 1315 bp sequence includes a region proximal to the transcription start site (5' TSS) and part of the 5' untranslated region of the C1ql2 mRNA (5' UTR). The sequence is rich in putative transcription factor binding sites (CpG islands). **b** Expression of GFP controlled by the C1ql2 promoter targets mature Mfs labelled by an antibody against synaptoporin. **c** *Top panels*, scheme of a bicistronic DNA construct that allows the co-expression of ChIEFtom and GFP under the control of the C1ql2 promoter. *Middle panels*, pictures showing the co-expression of ChIEFtom and GFP in the DG. Small squares indicated in the merge panel are presented enlarged in the lower row of panels. *Lower panels,* images acquired using a confocal microscope with a 20x or 60x objective showing that the two proteins ChIEFtom and GFP are well expressed in all cell compartments (dendrites, GCs soma, Mf bundles, Mf boutons in the stratum lucidum (SL). The fluorescence of mTomato fused to membrane bound ChR2 is prominent in dendrites and fibers whereas the fluorescence of cytosolic GFP is prominent in soma. The two polypeptides are well separated as shown by the expression of the membrane-bound ChIEFtom at the GC cell surface and of the free GFP in the cytosol (panel "gc: granule cells). However, infected neurons do not display any sign of degeneration, toxicity or aggregates of ChR2. **d** Western blots of ChIEFtom2A-Cre constructs (right) showing that ChIEFtom is fused to the remaining 2A peptide while Cre recombinase is free. **e** Expression of Cre recombinase under the control of the C1ql2 promoter allows the recombination of floxed-tdTomato gene in Ai9 mouse line leading to the expression of free cytosolic tdTomato in GC soma. **f** Western blots of samples from neuronal cultures prepared from PS1floxed embryos. Neurons were non-infected (Ø), infected with GFP- or with CreGFP-expressing viruses. Cre prevents most of PS1 protein expression. **g** Electrophysiological properties of ChIEF-expressing GCs studied in the whole-cell current clamp mode. A single action potential (AP) is evoked by a light pulse at 470 nm of 1 ms. A long burst of 100 stimuli at 20 Hz for 5 s triggers a long train of APs without failure. **h** Representative traces of single AP evoked in GC at increasing light intensity, and plot of the percentage of GC firing in function of light intensity. 1 ms pulses of 470 nm light were shined on top of GC using a 63x objective. Light intensity was increased up to 2 mW/cm2 (100%). **i** Properties of the GCs spikes in function of the intensity of light pulses. Plots of the threshold, amplitude and half-width of the GC action potential (GC-AP) are depicted. **j** Plot of EPSC amplitudes in function of light intensity recorded from 20 CA3 pyramidal cells. 1 ms pulses of 470 nm light were shinned on top of GC dendrites using a 63x objective. Light intensity was increased up to 2 mW/cm2. **k** Comparison of averaged values of Mf-EPSC amplitude, failure rate, paired-pulse and frequency facilitation recorded in CA3 pyramidal cells when stimulating Mfs with an extracellular electrode placed in the hilus or by stimulating GC dendrites with light. **l** Coefficient of variability (C.V.) of the amplitude of Mf-CA3 EPSCs recorded at 0.1 Hz in WT and PSKO conditions. **m** Representative Mf-EPSCs recorded in CA3 pyramidal cells in control conditions or in presence of 32 μM Cadmium^{2+} (Cd^{2+}). The presence of Cd^{2+} dramatically increases the failure rate and decreases the amplitude of the EPSCs. Errors bars represent s.e.m.

Supplementary figure 2

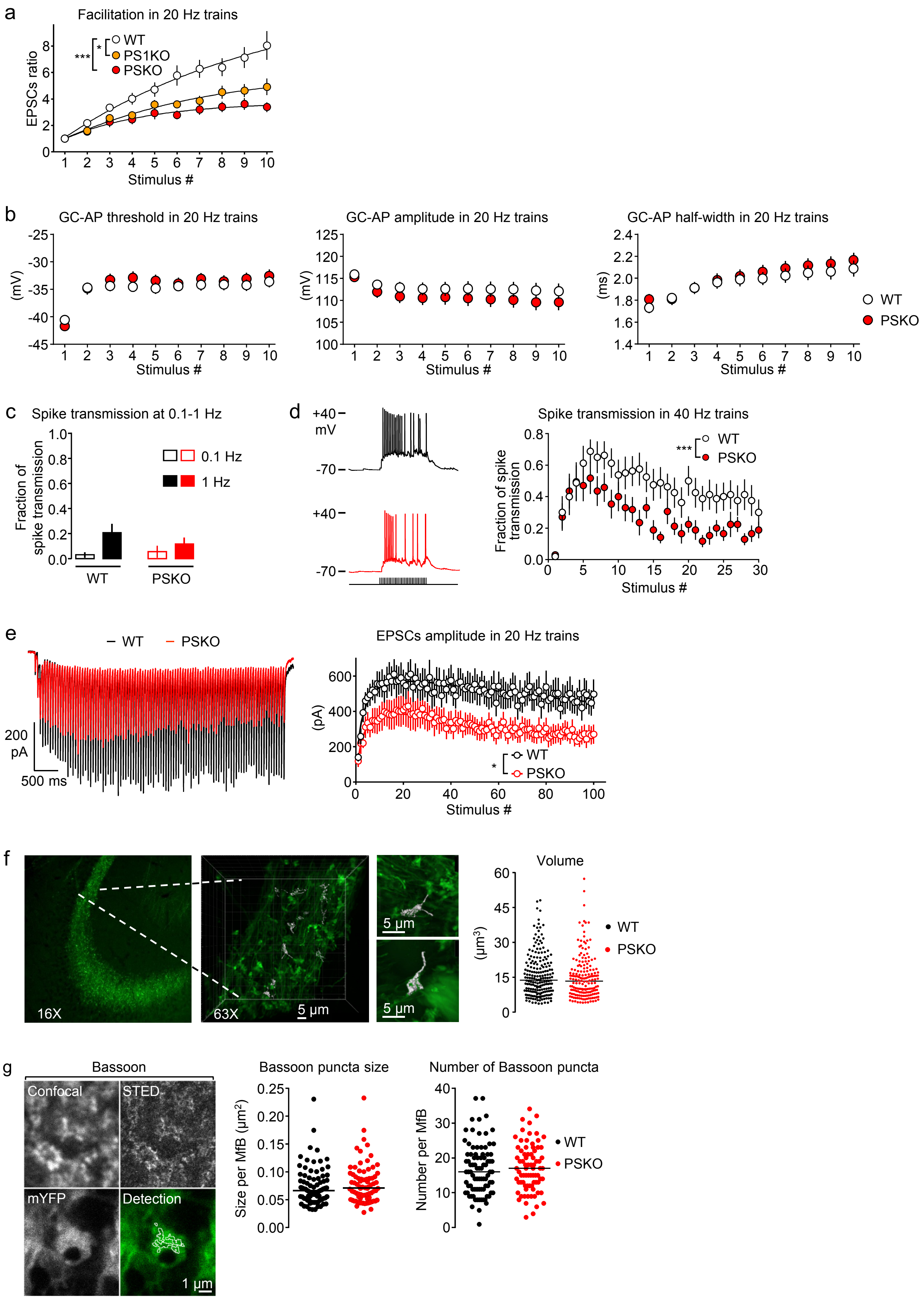

Supplementary figure 2 Electrophysiological properties of GCs, Mf-CA3 neurotransmission and Mf boutons morphology. **a** Analysis of Mf-EPSCs recorded in CA3 pyramidal cells while stimulating with ten repetitive light pulses at 20 Hz in WT, PS1KO and PS1/PS2KO conditions. Facilitation is represented by the ratio of EPSC amplitudes relative to the amplitude of the EPSC evoked by the first stimulus. Note that facilitation is impaired in the absence of PS1 alone. Two-way ANOVA, $p=0.0304$. **b** Properties of GCs spikes evoked by 10 light pulses of 1 ms at 20 Hz on GCs in WT and PSKO conditions. Plots of the threshold, amplitude and half-width of the GC action potential (GC-AP) are depicted. **c** Histograms of the mean fraction of spike transmitted in CA3 pyramidal cells recorded in the current-clamp mode at 0.1 or 1 Hz in WT and PSKO conditions. Note that in average the fraction of spike transmitted is low in all conditions even if a few neurons do fire quite reliably at 1 Hz. **d** Representative traces of APs in CA3 pyramidal cells in response to the stimulation of GC dendrites at 40 Hz in WT (black) and PSKO (red) conditions. Plot of the fractions of spike transmission (for 16 recorded neurons per genotype; 5 sweeps per neuron) in WT or PSKO genotype is shown. Note that at 40 Hz the highest rate of transmission is reached around 5 stimuli and that the plot in PSKO is lower than in WT condition (Two-way ANOVA, $p<0.0001$). **e** Representative Mf EPSCs obtained by illuminating the dendrites of GCs with 100 stimuli at 20 Hz. After the peak reached within ten stimuli, the amplitudes of Mf-EPSCs decrease while the charge of the train increases. The raw amplitude of Mf-EPSCs is plotted against the stimulus number. Note that Mf-EPSC amplitudes in PSKO condition are lower than in WT. Two-way ANOVA, n=20/genotype, $p<0.05$. **f** Confocal images of the *stratum lucidum* region of the hippocampus in brain slices where the Mfs projects onto CA3 pyramidal cells. A zoom in using a 63x objective allows to observe Mf boutons. 3D reconstruction of some Mf boutons after acquisition of Z-stack are depicted in grey. A zoom on two large Mf boutons allows to appreciate their large volume and complex structure. The scatter plots with medians of the volume of Mf boutons in WT and PSKO conditions are represented. **g** Evaluation of Bassoon puncta number and surface using STED microscopy. A representative image of a Mf bouton labelled with membrane-bound YFP (mYFP, bottom left panel) and stained against Bassoon (upper left panel in confocal; upper right panel in STED microscopy) is shown. Detected Bassoon positive puncta are delimited with white lines. Scatter plots of average size and the number of Bassoon puncta per Mf bouton are depicted. 76 Mf boutons have been analyzed per genotype. The absence of PS does not impact on the number of putative releasing sites. Errors bars represent s.e.m.

Supplementary figure 3

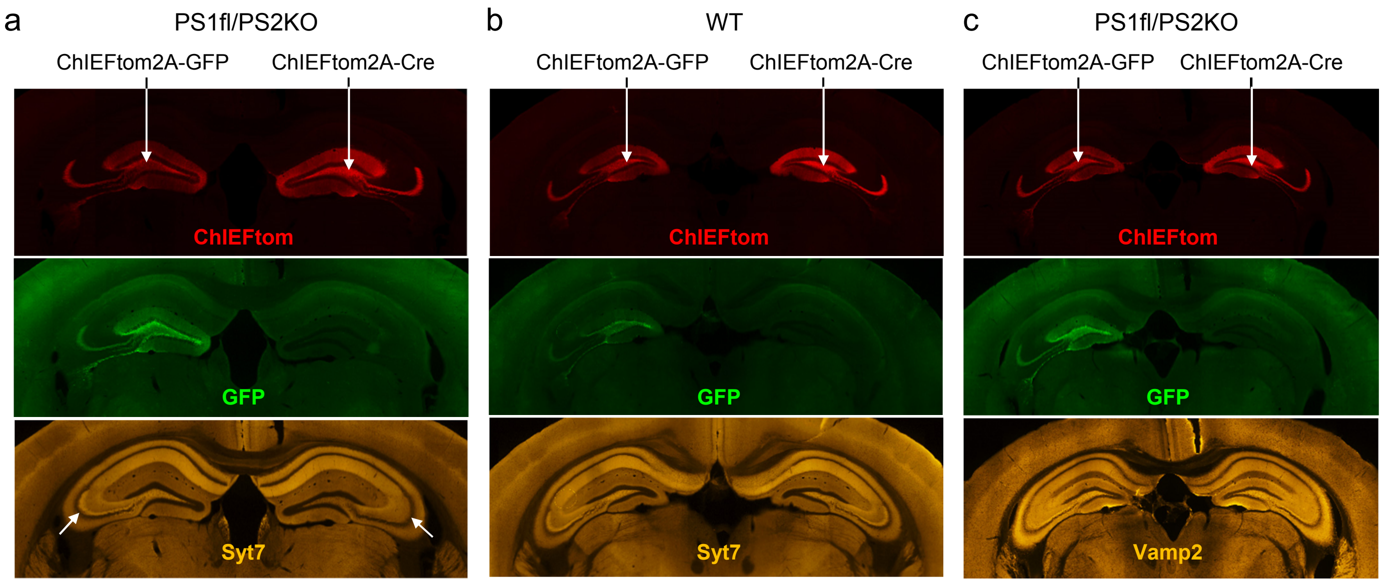

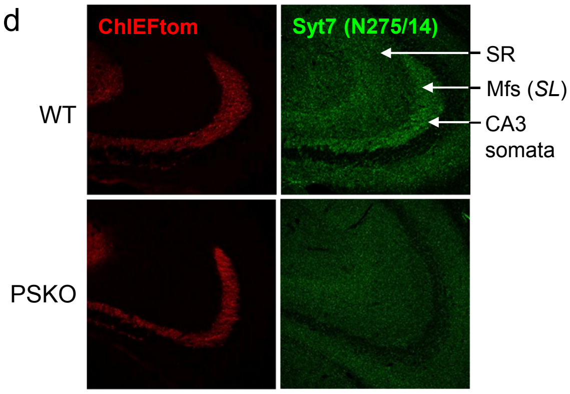

Supplementary figure 3 Effect of targeted deletion of PS in GCs on Syt7 and Vamp2 expression. **a-c** Pictures of coronal sections from mice injected in the left hemisphere with a LV expressing ChIEFtom and GFP and on the right hemisphere with a LV expressing ChIEFtom and Cre. **a**, in PSfl1/PS2KO mice, a decrease of Syt7 staining is observed only in the SL where PS1 has been removed (compare the two oblique arrows). In WT condition stained for Syt7 (**b**) or in PSfl1/PS2KO mice stained for Vamp2 (**c**), no difference is observed between the two hemispheres. Note that the absence of PS2 alone does not noticeably affect the staining of Syt7 compared to WT (compare the left hemispheres of WT and PS1fl/PS2KO conditions). **d** Confocal images of the *stratum lucidum* (SL) where the Mfs can be distinguished in the red channel (ChIEFtom) and stainings of Syt7 depicted in green. The Syt7 staining in PSKO Mfs was negligible in comparison to WT and indicates that PS supports the expression of Syt7.

Supplementary figure 4

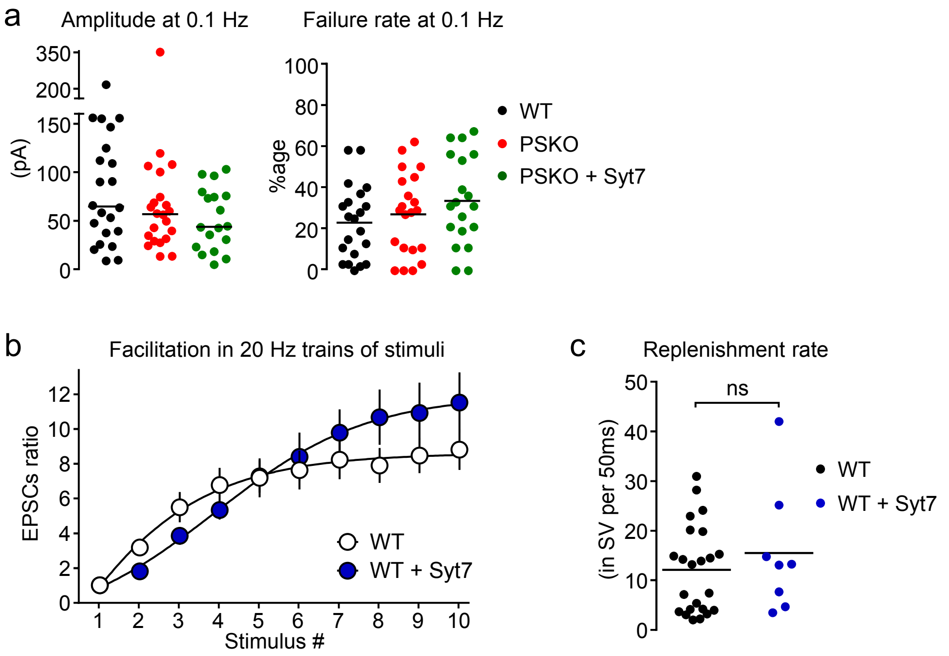

Supplementary figure 4 Basal transmission at PSKO Mf-CA3 synapses rescued for Syt7. **a** Basal transmission at Mf-CA3 synapses in WT, PSKO and PSKO+Syt7 conditions. Scatter plots with medians of the amplitude, and means of the failure rate of EPSCs recorded in CA3 neurons at 0.1 Hz. **b** Analysis of presynaptic facilitation in 20 Hz trains. Facilitation is represented by the amplitude of the EPSCs normalized to the amplitude of the first EPSC. The over-expression of Syt7 in WT does not significantly alter presynaptic facilitation. n=24 in WT, 14 in WT+Syt7. **c** Scatter plots with means of replenishment rates at 20 Hz calculated neuron per neuron are represented by genotype. The replenishment rate of WT+Syt7 condition does not differ from the WT condition. Errors bars represent s.e.m.

Supplementary figure 5

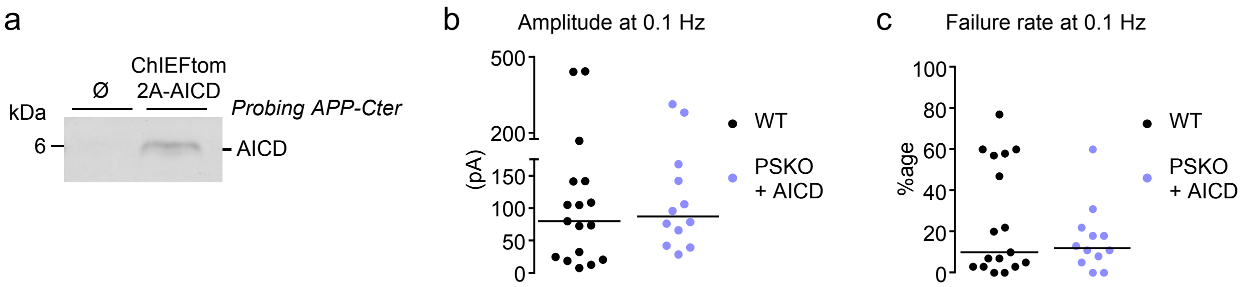

Supplementary figure 5 Basal transmission at PSKO Mf-CA3 synapses with rescue of AICD. **a** Western blots of (LoxStopLox)-ChIEFtom2A-AICD construct (right) showing the expression of AICD at the protein level. **b** Basal transmission at Mf-CA3 synapses in WT and PSKO with re-expression of AICD. Scatter plots with medians of EPSCs amplitude recorded in CA3 pyramidal cells in the whole cell voltage-clamp mode while stimulating GCs with light at 0.1 Hz. **c** Scatter plots with mean failure rates in conditions as in (**a**).

Supplementary figure 6

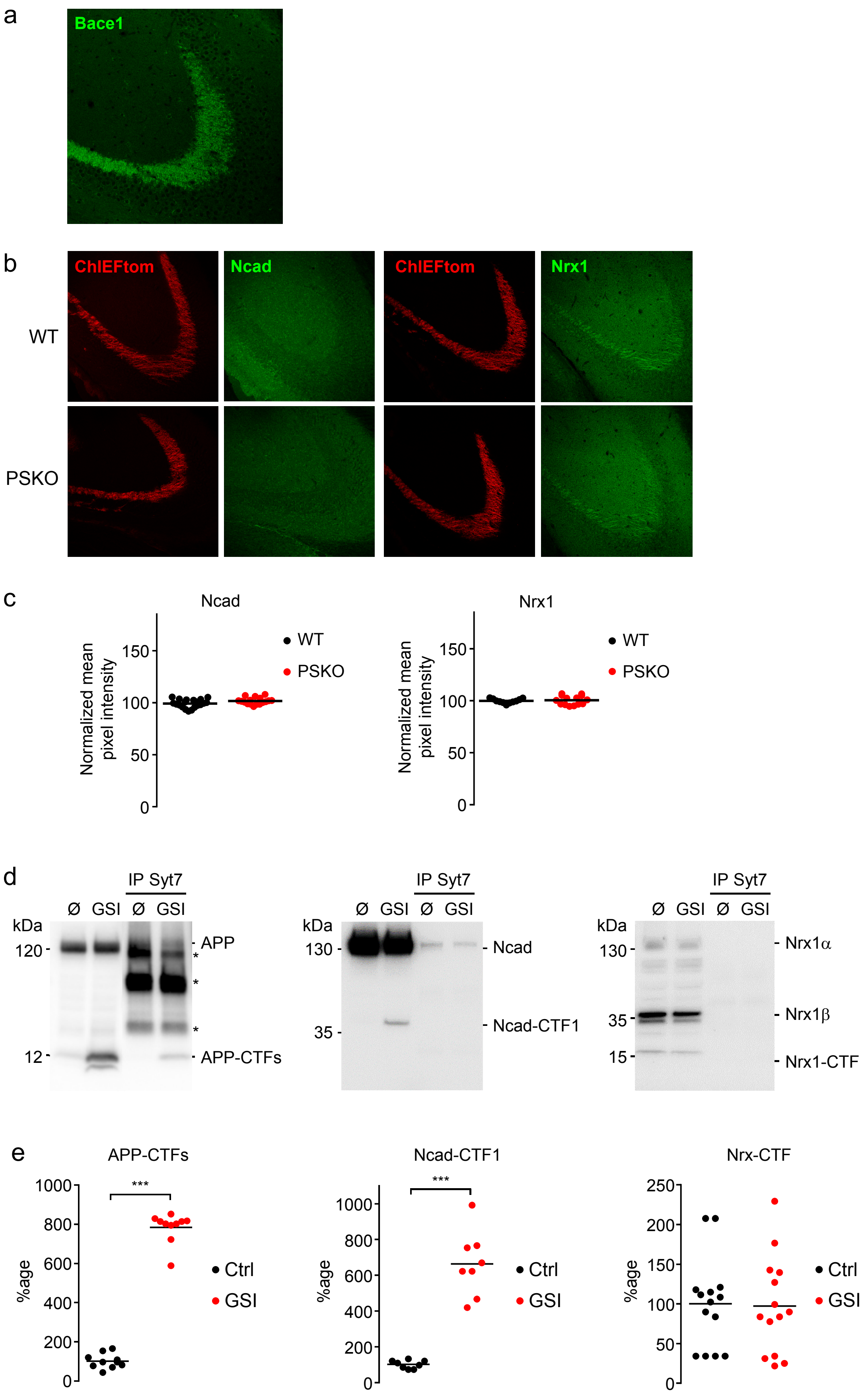

Supplementary figure 6 Expression of γ-secretase substrate candidates in Mfs and accumulation of C-terminal stubs upon γ-secretase inhibition. **a** Confocal images of the Mfs tracts in WT and PSKO conditions observable in the red channel (ChIEFtom) and green channel (staining for Ncadherin Cterminal (Ncad) or Neurexin Cterminal (Nrx1)). Mfs are faintly labelled by the Ncad antibody and more prominently by the Nrx1 antibody. The absence of PS does not alter labelling with these two antibodies. **b** Scatter plots of the mean pixel intensity in the *SL* normalized to the mean pixel intensity in the *stratum radiatum* for Ncad and Nrx1 labelling. **c** The absence of PS does not increase the intensity of the labelling indicating that Cterminal stubs of Ncad or Nrx1 do not accumulate in Mfs. **d** Western-blot of protein extracts from neuronal cultures treated or not with a γ-secretase inhibitor (GSI). GSI induces the accumulation of APP-CTFs and Ncad Cterminal stub (Ncad-CTF1) but not of the band expected to be Neurexin1-CTF (16 kDa). IP of Syt7 pull down APP-FL, APP-CTFs and Ncad but not Ncad-CTF1 or Neurexin1 of any form. **e** Quantitative analysis of the CTF bands detected in western blot of starting materials from neuronal cultures treated or not with GSI as in (**d**). An increase of 800% in the expression of APP-CTFs was detected. An increase of 650% in the expression of Ncad-CTF was detected. The expression level of Neurexin-CTF was unchanged by treatment with GSI.

Supplementary figure 7

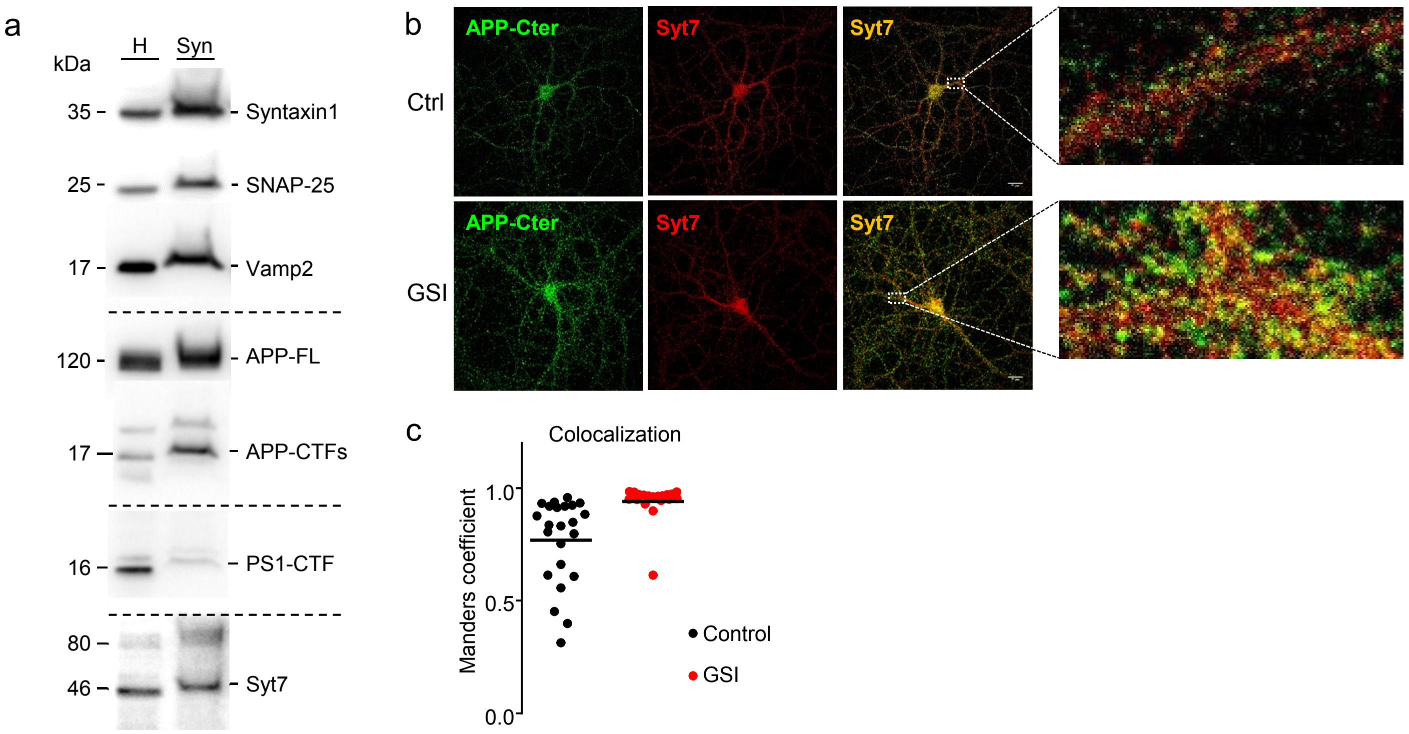

Supplementary figure 7 Characterization of synaptosomal protein pattern and colocalization between Syt7 and APP in neurons. **a** Characterization by WB probing of synaptosomal protein lysate (Syn) versus protein lysate from the crude homogenate (H) of adult mouse brain. 'Syn' is characterized by an enrichment in Syntaxin1a and SNAP-25. While PS1 is detectable, APP (both FL and CTF) and Syt7 are abundant in 'Syn'. **b** Confocal images of APP-Cter (green channel) and Syt7 (red channel) in neuronal cultures treated (GSI) or not (Ctrl) with GSI. Zoom-in representations are depicted in the right. APP colocalizes with Syt7 in both conditions. **c** The extent of colocalization of images as in (**b**) is assessed using the Manders coefficient (see Methods). The colocalization is higher in neurons treated with GSI.

Supplementary figure 8

Supplementary figure 8 Original blots shown in full molecular weight range.

Figure 3d (Syt7)

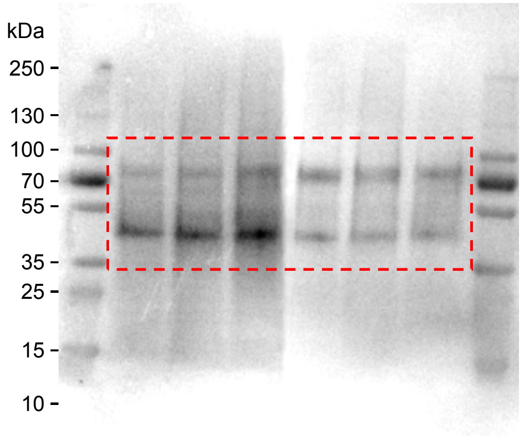

Figure 5a (Syt7)

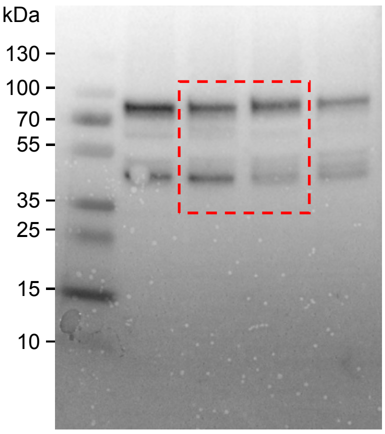

Figure 5b (Vamp2)

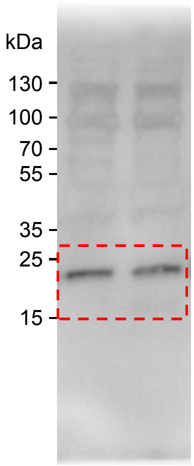

Figure 5b (Syt1)

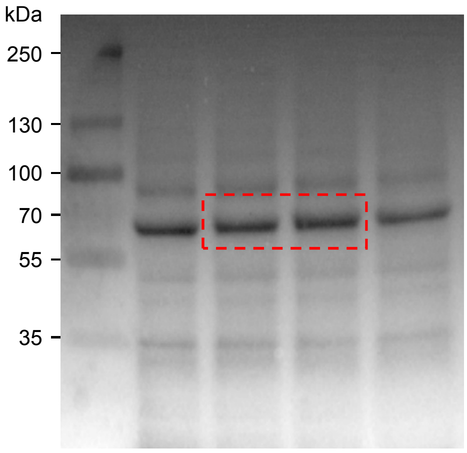

Figure 5b (Syntaxin1)

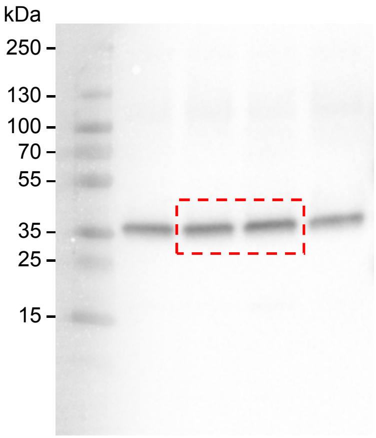

Figure 6e (APP-FL and CTFs)

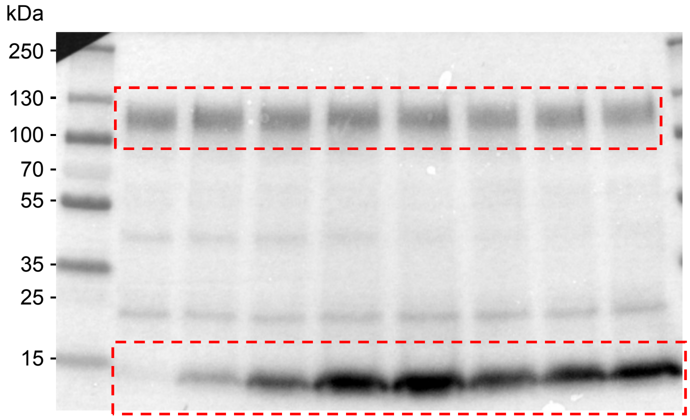

Figure 6e (Syt7)

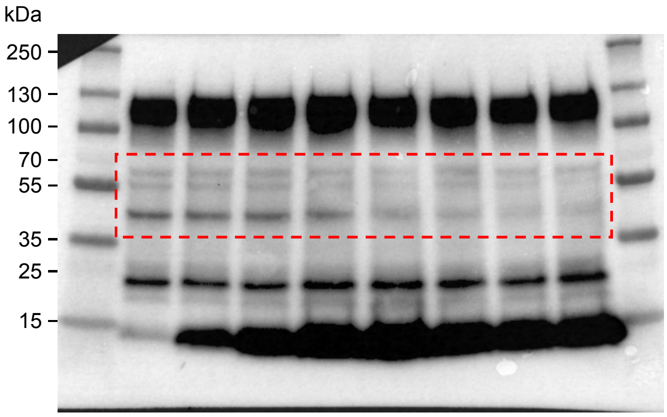

Supplementary figure 8

Figure 7d (APP-FL)

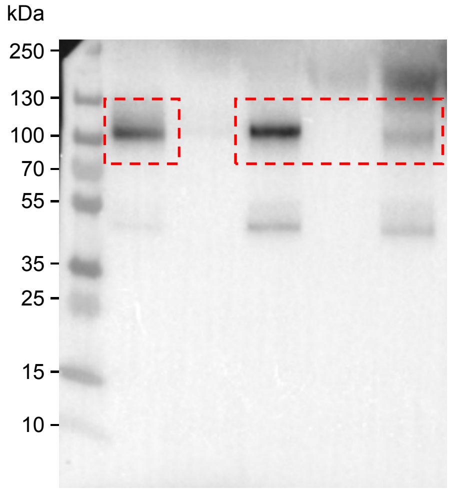

Figure 7d (Syt7)

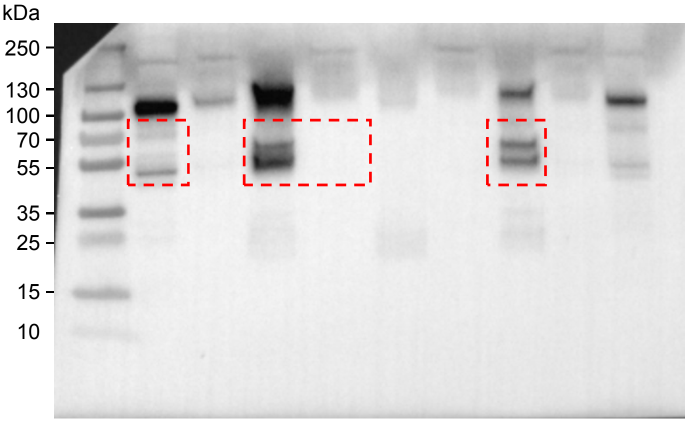

Supplementary figure 1d
(2A)

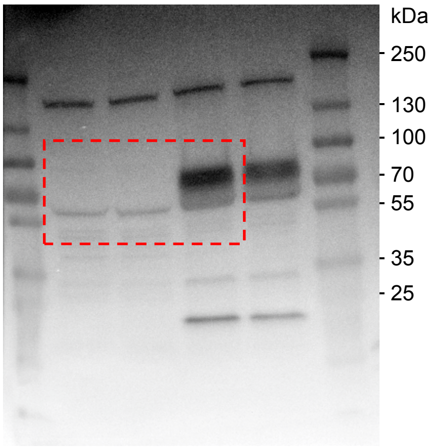

Supplementary figure 1d
(tomato)

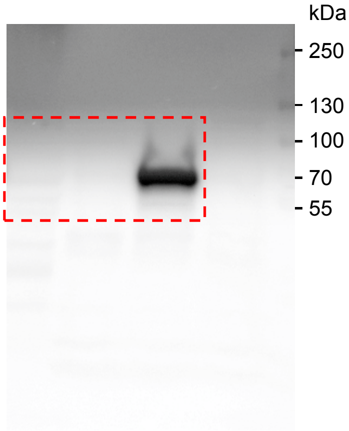

Supplementary figure 1d
(Cre)

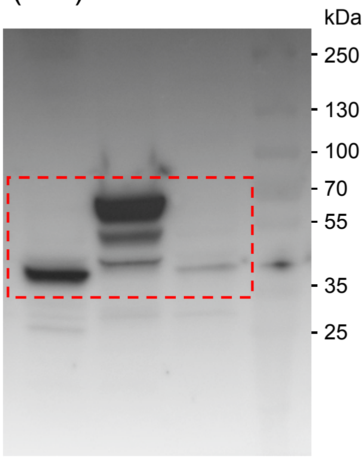

Supplementary figure 1f
(GFP)

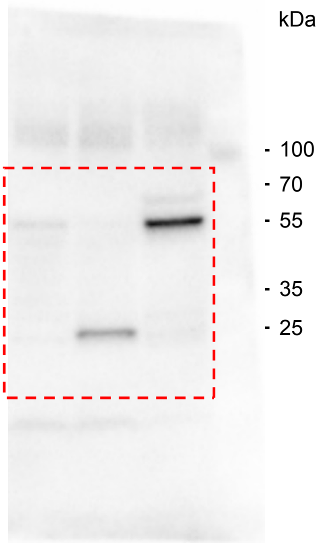

Supplementary figure 1f
(Cre)

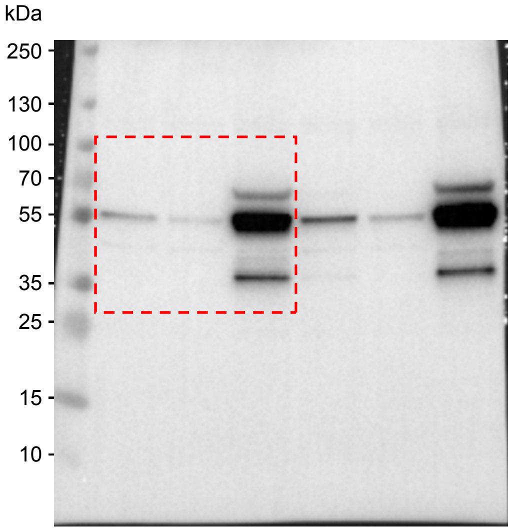

Supplementary figure 1f
(PS1-CTF)

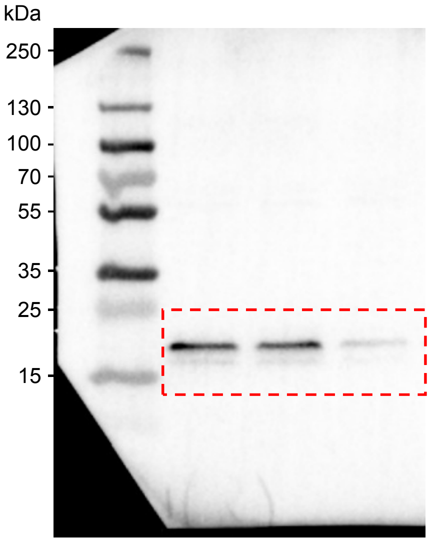

Supplementary figure 8

Supplementary figure 5a (APP, AICD)

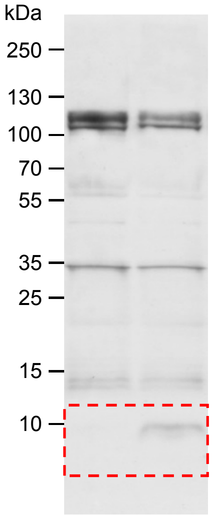

Supplementary figure 6d (APP, AICD)

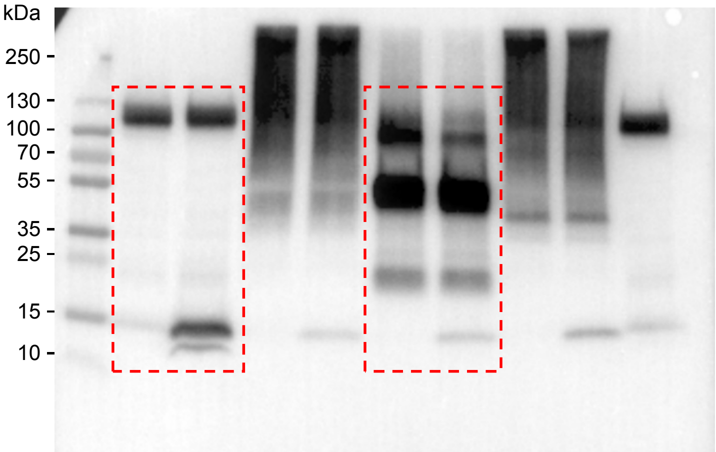

Supplementary figure 6d (Ncad)

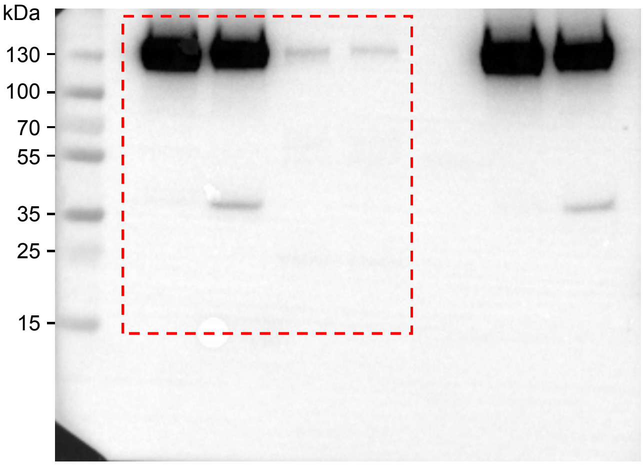

Supplementary figure 6d (Nrx)

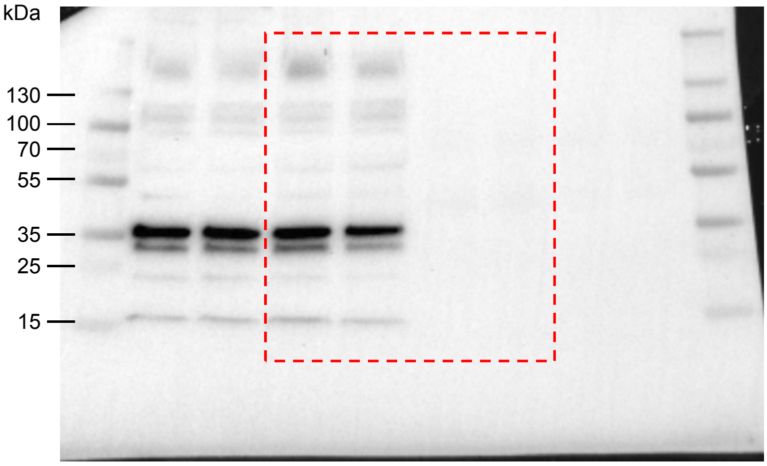

Supplementary figure 8

Supplementary figure 7a (Syntaxin)

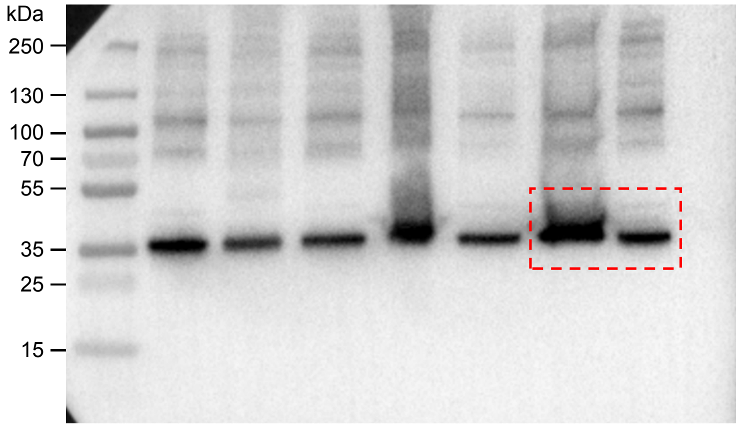

Supplementary figure 7a (SNAP25)

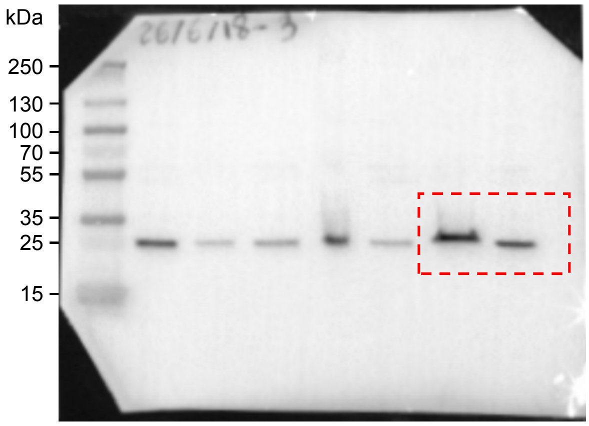

Supplementary figure 7a (Vamp2)

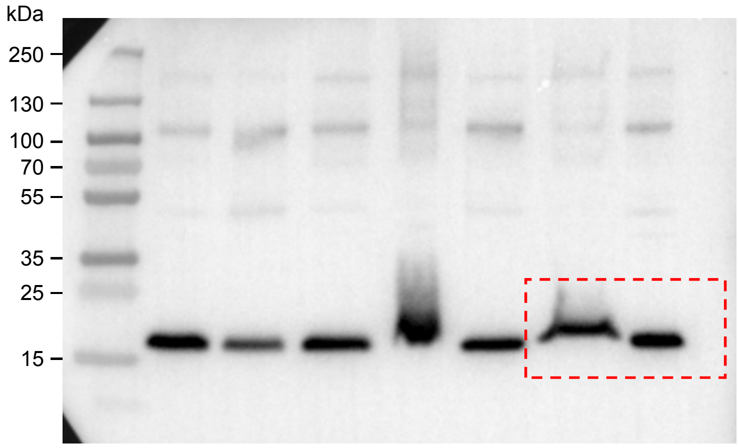

Supplementary figure 7a (APP)

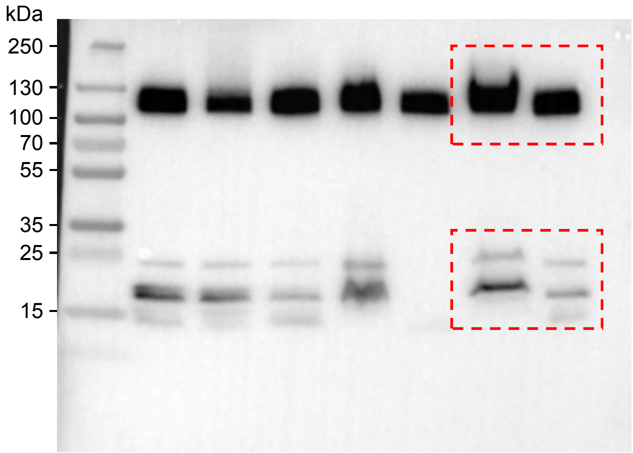

Supplementary figure 7a (PS1-CTF)

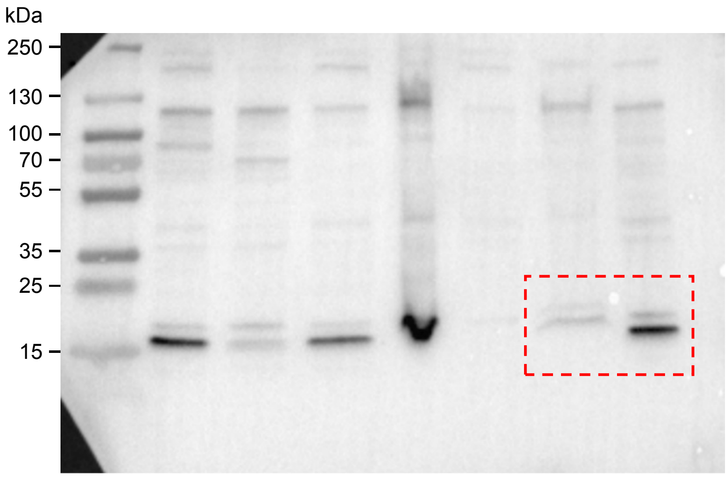

Supplementary figure 7a (Syt7)

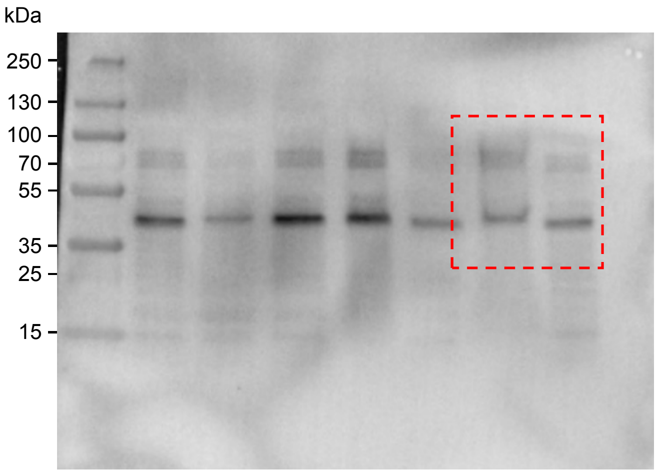

Supplement: Supplementary file 1 — Peer Review File [file 41467_2018_6813_MOESM1_ESM.pdf]
